# Concurrent Diagnosis and Management of Primary Mediastinal Germ Cell Tumor and Klinefelter Syndrome

**DOI:** 10.7759/cureus.85003

**Published:** 2025-05-28

**Authors:** David D Kim, Juan M Alcantar, Fukai L Chuang

**Affiliations:** 1 Hematology and Medical Oncology, University of California Los Angeles David Geffen School of Medicine, Los Angeles, USA

**Keywords:** germ cell tumor, hypogonadism, klinefelter syndrome, mediastinal mass, primary mediastinal germ cell tumor, testosterone replacement therapy, vip chemotherapy

## Abstract

Primary mediastinal nonseminomatous germ cell tumor (PMNSGCT) stands out among other germ cell tumors not only because of its mediastinal site of origin, but by its aggressive behavior, need for maximal multidisciplinary therapy, and relatively poor prognosis. We present a case of a 22-year-old man who was evaluated for chest-related and systemic symptoms and found to have a large mediastinal mass compressing the pulmonary artery with no apparent disease outside the mediastinum. Physical exam at presentation to medical oncology revealed gynecomastia and small testicles, suspicious for sex hormone dysregulation. Workup revealed the diagnosis of PMNSGCT as well as Klinefelter syndrome (KS), a sex chromosome abnormality condition in males caused by an extra copy of the X chromosome. The association between these two conditions is reported but not well known, given the uncommon incidence of both and the lack of an obvious connection. Pre-treatment management included referral to multidisciplinary specialties and assessment for fertility preservation. The patient was treated with a curative intent with etoposide, ifosfamide, and cisplatin (VIP) chemotherapy for four cycles at full doses despite various side effects. He underwent surgical resection of his residual mediastinal mass, with pathology revealing a complete pathologic response, which is considered a good prognostic indicator for survival. Therefore, he did not require adjuvant chemotherapy. He was started on testosterone replacement therapy for KS-related hypogonadism with improvement in his testosterone level and quality of life. To date, he has not had evidence of recurrence. We discuss the evaluation and management of PMNSGCT and KS, as well as the evidence for their association reported in the literature.

## Introduction

Testicular germ cell tumor is the most common cancer that develops in young adult men [1] and is generally known to have a good prognosis, curable even when metastatic. Germ cell tumors are divided into seminomas and nonseminomas based on microscopic morphology, and the former being particularly sensitive to chemotherapy, with long-term survival rates of up to 90%. Although the majority of male germ cell tumors originate in the testes, about 5% of cases have extragonadal disease without a testicular primary, including in the mediastinum (most common extragonadal site), retroperitoneum, or pineal gland [2]. Primary mediastinal nonseminomatous germ cell tumor (PMNSGCT) is a unique subtype of germ cell tumors with various distinct features compared to its gonadal and other extragonadal germ cell tumor counterparts. The aggressive nature of PMNSGCT is evident by the long-term survival rate of only 30% cited by early reports [2]. The understanding, multidisciplinary treatment, and prognosis of PMNSGCT have improved over time, but the disease remains a challenge in oncology.

Klinefelter syndrome (KS) was first described in 1942 by Klinefelter et al. [3] in a report of nine men with common features of small testicles, gynecomastia, azoospermia, and primary hypogonadism. The underlying genetic basis for KS was not discovered until 1959 when it was associated with a 47, XXY karyotype. Despite many decades having passed since our early understanding of KS, awareness of KS by the general medical community remains elusive, and only 25% of cases are given the right diagnosis, usually during adulthood [4]. The discovery that KS can cause significant morbidity and mortality has made early detection and management of KS imperative.

We present a case of concurrent diagnosis and management of PMNSGCT and KS. Although these two conditions do not appear related on the surface, there is a body of evidence supporting a strong association with an underlying embryologic explanation. Our case highlights that knowledge of PMNSGCT and KS, along with their association and attention to physical exam findings, can lead to timely diagnosis, proper management, and good outcomes for both conditions.

## Case presentation

A 22-year-old man was referred to the medical oncology clinic for evaluation of a mediastinal mass. He had presented to his primary care physician with 40 lb. unintentional weight loss, drenching night sweats, persistent cough, chest discomfort, and dyspnea on exertion. A chest X-ray ordered by his primary care physician revealed a large left hilar mass. CT chest, abdomen, and pelvis with IV contrast confirmed a soft tissue mass measuring 9.5 × 9.3 cm in the prevascular mediastinum, causing mass effect on the left pulmonary artery. There was no evidence of other masses or lymph node enlargement throughout the body. The patient had no significant past medical or surgical history. He denied a history of tobacco smoking. He was unmarried and had no children. There was no family history of cancer. Physical exam was only remarkable for gynecomastia and small testicles measuring 1.5 cm in greatest dimension without a distinct mass. Height was 6 feet 4 inches, and weight was 209 lb. The top differential diagnoses for his mediastinal mass were lymphoma or mediastinal germ cell tumor. Given his physical exam findings, sex hormone dysregulation was also suspected.

**Figure 1:**
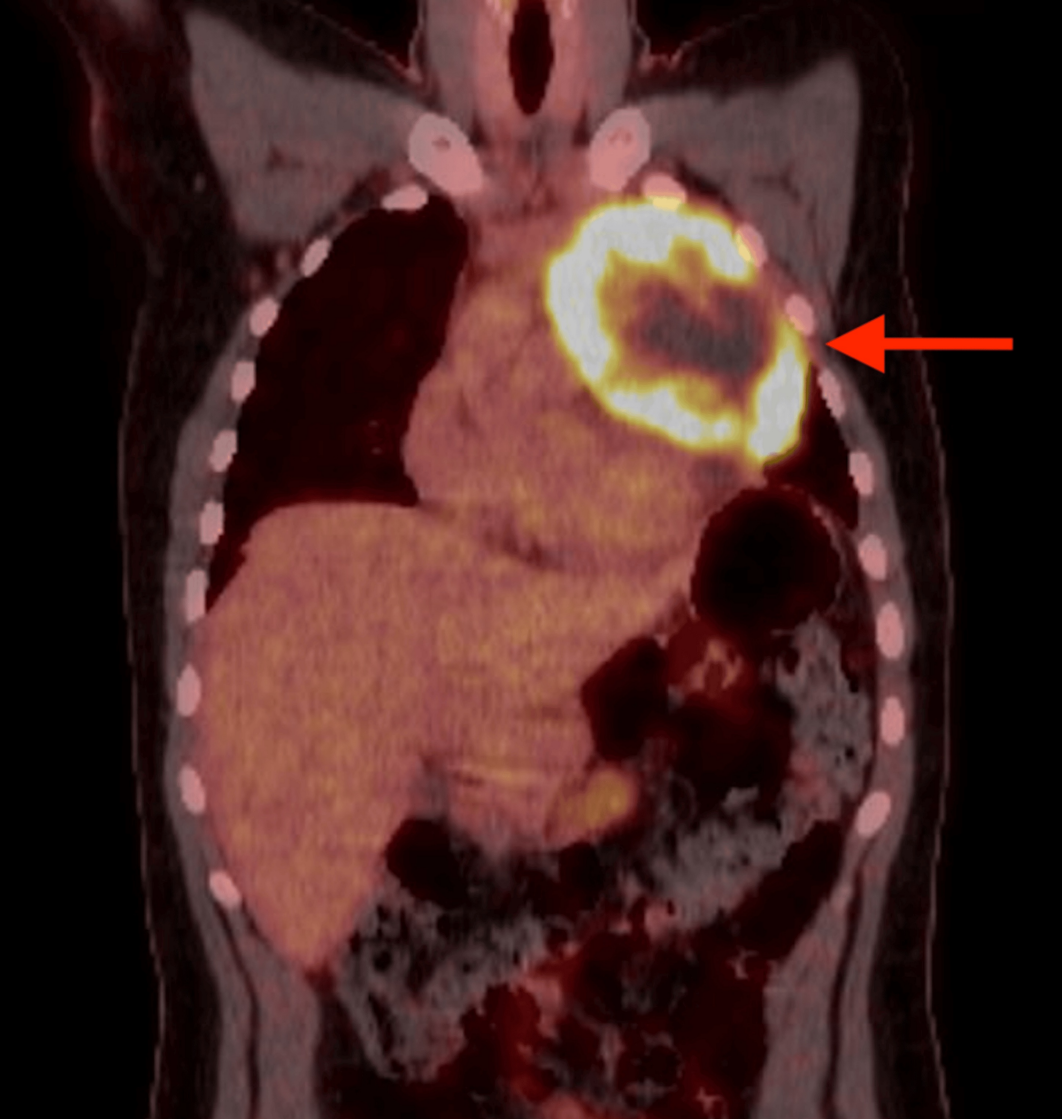
FDG PET-CT scan showing a left-sided anterior mediastinal mass with increased FDG uptake (arrow). FDG PET-CT: Fluodeoxyglucose Positron Emission Tomography-Computed Tomography

Evaluation ordered by medical oncology included the following: Laboratory studies showed significant elevation of alpha-fetoprotein (AFP), lactate dehydrogenase (LDH), and erythrocyte sedimentation rate (ESR), but beta human chorionic gonadotropin (hCG) was not significantly elevated. Testosterone level was low while follicle-stimulating hormone (FSH) and luteinizing hormone (LH) levels were high (Table 1). Scrotal ultrasound confirmed atrophic testicles without a mass. Fluorodeoxyglucose (FDG) PET-CT scan showed a PET-avid anterior prevascular mediastinal neoplasm measuring 11.8 cm in greatest dimension without evidence of distant metastasis (Figure 1). MRI of the brain did not show brain metastasis. A presumptive diagnosis of PMNSGCT was made. CT-guided mediastinal mass biopsy was performed, and pathology confirmed a malignant nonseminomatous germ cell tumor with a predominant yolk sac component. There was no evidence of sarcomatoid or teratomatous features. Fifty percent of the tumor was necrotic. He was referred to a thoracic surgeon, who ordered an MR angiogram of the chest, which revealed high-grade stenosis of the left pulmonary artery; early invasion could not be excluded. There was a small left pericardial effusion with findings suggestive of pericardial invasion.

**Table 1:** Pertinent Laboratory Data

Laboratory Test	Result	Reference Range
Alpha-fetoprotein (AFP)	12,905 ng/mL	0-6.7 ng/mL
Lactate dehydrogenase (LDH)	670 U/L	125-256 U/L
Beta human chorionic gonadotropin (hCG)	2 mIU/mL	<1 mIU/mL (Male)
Erythrocyte sedimentation rate (ESR)	85 mm/h	<=12 mm/h
Testosterone	86 ng/dL	200-1000 ng/dL (Adult male)
Follicle-stimulating hormone (FSH)	21.6 mIU/mL	1.6-9 mIU/mL (Adult male)
Luteinizing hormone (LH)	43.2 mIU/mL	2-12 mIU/mL (Ault male)

**Figure 2:**
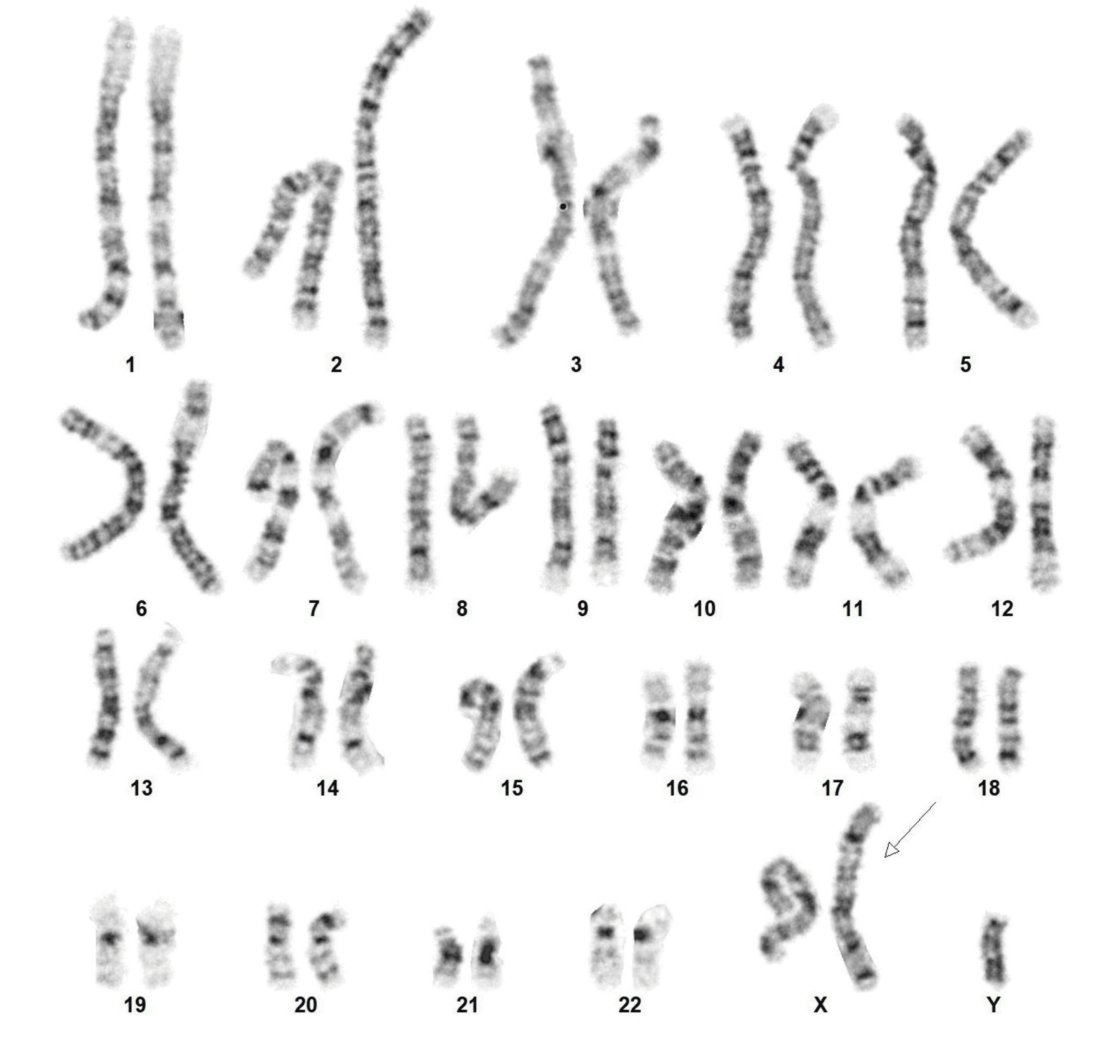
Karyotype showing an extra copy of chromosome X (arrow).

Given the diagnosis of PMNSGCT and primary hypogonadism, KS was suspected. Karyotype from a blood draw was ordered and confirmed an extra copy of the X chromosome in all cells analyzed (47, XXY), consistent with a diagnosis of KS (Figure 2). He was referred to genetic counseling, urology, and endocrinology. Semen analysis showed azoospermia. Urology offered to perform microsurgical testicular sperm extraction (microTESE) for cryopreservation of sperm prior to starting chemotherapy, but he declined, given that the chance of success was reportedly not high, and his desire to start chemotherapy immediately.

After discussion of his treatment options, which included the standard bleomycin, etoposide, and cisplatin (BEP) chemotherapy regimen, he was started on etoposide (VP-16), ifosfamide, and cisplatin (VIP) chemotherapy. VIP was administered as an inpatient and given with pegfilgrastim to reduce the risk of neutropenic infection. His chemotherapy treatment course was complicated by recurrent syncopal episodes. Echocardiogram showed a preserved ejection fraction. There was compression of the left pulmonary artery and left superior pulmonary vein by the mass, but no evidence of main right ventricle outlet obstruction on cardiac MRI. Cardiology and neurology believed he was having vasovagal syncope. He also developed recurrent stomatitis, dehydration, pancytopenia requiring blood and platelet transfusions, and neutropenic fever with negative blood and urine cultures during his treatment cycles. Despite these side effects, his chemotherapy dose was not reduced per the patient’s request since he was being treated with a curative intent, and dose reduction might compromise his chance of cure. His AFP and LDH levels dropped with each cycle of VIP chemotherapy. By four cycles of VIP, his AFP and LDH levels were within normal range. The repeat chest CT with IV contrast showed a significantly smaller but residual mass abutting the left pulmonary artery, left superior pulmonary vein, and aortic arch.

After recovering from chemotherapy, the patient underwent resection of the anterior mediastinal mass, thymectomy, en bloc pericardial resection with bovine pericardial reconstruction, and left upper lobe lung wedge resection. Surgical pathology reported no viable malignancy, consistent with a complete pathologic response. There were tumor necrosis, foamy histiocytes, and fibrosis in the tumor bed. Several regional lymph nodes were not involved by malignancy. He recovered well from surgery and did not need additional adjuvant chemotherapy, given his complete pathologic response. Endocrinology started the patient on testosterone replacement therapy, leading to normalization of his testosterone level. He gained back the weight he had lost from his cancer and cancer therapy, and reported improvement in his energy. Surveillance with tumor marker levels and CT imaging has not shown any signs of cancer recurrence. He remains with no evidence of disease and lives an active life at the time this case report was written, 2 years since his diagnosis.

## Discussion

This case provides an opportunity to review the presentation, evaluation, management, and prognosis of PMNSGCT and KS, as well as the evidence for their association.

According to the International Germ Cell Consensus Classification (IGCCC) [1], PMNSGCT is classified in the poor-risk group with the worst prognosis, along with nonseminomatous germ cell tumors with nonpulmonary visceral metastases (such as brain, liver, or bone involvement) or with exceptionally high serum tumor marker levels. PMNSGCT develops in the anterior mediastinum and is often undetected until it has reached a considerable size and has involved adjacent mediastinal structures. The most common presenting symptoms are dyspnea, chest pain, cough, fever, and weight loss [2]. This is important to note as fever, weight loss, and an anterior mediastinal mass can be the presentation of primary mediastinal lymphoma with B symptoms. A presumptive diagnosis of PMNSGCT can be made based on CT scan findings and elevated serum tumor markers (alpha-fetoprotein (AFP), beta human chorionic gonadotropin (HCG), and lactate dehydrogenase (LDH)) in most cases, as was for our patient. Any elevation of AFP rules out pure seminoma and categorizes the tumor as nonseminoma. However, histologic diagnosis is generally recommended before initiation of chemotherapy. PMNSGCT biopsy pathology can reveal a mixture of different histologies, including yolk sac tumor, embryonal carcinoma, choriocarcinoma, and teratoma. Staging should include CT imaging of the body as well as MRI of the brain to assess for metastases. Kesler et al. [5] reported that 96% of patients with PMNSGCT had elevated serum tumor markers, and 34% had metastatic disease at diagnosis.

The mainstay of treatment for PMNSGCT is cisplatin-based chemotherapy. BEP for four cycles has been the standard of care for intermediate-risk and poor-risk germ cell tumors, including PMNSGCT, and has vastly improved outcomes compared to the pre-cisplatin era. Unfortunately, bleomycin is well-known to cause pulmonary toxicity, especially in patients with prolonged exposure to high concentrations of oxygen. Patients with PMNSGCT undergo lengthy thoracic surgery and were found to have an increased incidence of respiratory failure and acute respiratory distress syndrome (ARDS) after treatment with BEP. Therefore, the VIP regimen has emerged as an alternative regimen for patients who are at risk for bleomycin-related pulmonary toxicity, and four cycles of VIP are recommended for PMNSGCT by many experts. Kessler et al. [5] from Indiana University reported a historical reduction in postoperative respiratory failure rate to 2.6% in patients treated with VIP, compared to 14.8% in those treated with BEP. All postoperative deaths occurred in patients exposed to bleomycin, compared to no postoperative deaths in patients treated without bleomycin. VIP is given over 5 days, typically as an inpatient, and produces more hematologic toxicity than BEP, which is an outpatient regimen [6].

Surgical resection of radiographic residual disease after chemotherapy is also recommended and has improved outcomes. Preoperative elevation of serum tumor markers is evidence of active residual disease and a poor prognostic marker, but should not preclude surgery. Most patients with PMNSGCT are able to undergo resection even when the tumor is locally advanced, except in the uncommon cases of extensive involvement of the great arteries or middle mediastinal involvement. Reconstruction of vasculature is performed when feasible. Surgical pathology can show complete tumor necrosis (indicating complete pathologic response to chemotherapy), residual teratoma, or other malignant nonseminoma. Although long-term survival for PMNSGCT is reported to be around 40-50% with current treatment protocols, those that achieve complete tumor necrosis can expect a 5-year survival rate as high as 90% [5]. Therefore, the patient in our case is expected to have a good outcome. In contrast, those with residual nonseminoma on surgical pathology have significantly inferior outcomes. Two additional cycles of adjuvant chemotherapy are given for patients with pathologic residual disease and negative serum tumor markers, whereas adjuvant chemotherapy is not necessary for complete pathologic responders. Surveillance for recurrent disease involves periodic monitoring of serum tumor markers and chest imaging at gradually longer intervals over five years, then annually. Salvage therapy is offered to patients with refractory or relapsed PMNSGCT and usually includes high-dose chemotherapy and autologous stem cell transplant. Although long-term survival rates were reported to be less than 10% in relapsed PMNSGCT treated with salvage chemotherapy regimens [2], updated data on high-dose chemotherapy and autologous stem cell transplant showed 2-year progression-free survival and overall survival of over 30% [7].

The association between PMNSGCT and KS was first reported in small case reports. Then, in 1987, a study by Nichols et al. [8] described 22 patients with primary mediastinal germ cell tumors for whom chromosome analyses were performed. Twenty-two percent of these patients were found to have KS, all with nonseminoma subtypes. The median age of these patients with KS was younger than those without KS (15 vs 28 years). Based on these findings, the authors concluded that KS predisposes to PMNSGCT. Other case series have supported this strong association, reporting 8-18% incidence of KS in primary mediastinal germ cell tumors [2]. Therefore, experts have recommended screening for KS in all patients diagnosed with PMNSGCT. Based on the incidence of PMNSGCT in the general population, males with KS have a relative risk of greater than 50 of developing PMNSGCT, although the absolute risk remains small given the rarity of PMNSGCT [9,10]. Although screening for PMNSGCT in KS males has not been recommended, KS males should be educated on their health risks, and clinicians should have a high index of suspicion for PMNSGCT in KS males who present with symptoms of PMNSGCT or are found to have a mediastinal mass.

Pathogenesis of PMNSGCT in KS is believed to be related to residual germ cells in the mediastinum left behind during their migration from the yolk sack endoderm to the gonads along the urogenital ridge during embryogenesis [2,8]. This migration of germ cells occurs along the midline and can leave undescended cells that can also develop into germ cell tumors of other extragonadal sites, such as the pineal gland and retroperitoneum. The high levels of gonadotropins (LH and FSH) that develop with testicular degeneration in KS are believed to cause chronic stimulation of these extragonadal germ cells. This chronic stimulation, combined with the possible increased malignant potential of KS germ cells, can eventually lead to oncogenesis in some.

The significance of diagnosing KS in a previously undiagnosed male is manifold. KS is the most common sex chromosome abnormality in males, with an incidence of 150 out of every 100,000 male births [4]. The karyotype 47, XXY is found in 85-90% of KS, with the rest having abnormalities such as XXY mosaicism or more than one extra X chromosome, and occurs from nondisjunction during paternal or maternal gametogenesis. The classic presentation of KS includes atrophic testicles, gynecomastia, infertility, and hypergonadotropic hypogonadism. Physical exam universally reveals small testicles with a mean adult bi-testicular volume of 4-7 mL and a smaller size noticeable even before puberty, although less pronounced. Therefore, testicular exam can be used as a screening tool for KS, but unfortunately is not carried out by many physicians. Other physical exam findings, such as gynecomastia, tall stature, and increased waist circumference, are less consistent. Diagnosis of KS is made based on the presence of the KS phenotype and karyotyping. Adult diagnosis of KS usually occurs during workup for infertility. Only 10% of KS males are diagnosed before adulthood, leaving the majority undiagnosed or diagnosed late and missing opportunities for intervention. Early diagnosis and treatment of KS is imperative given its significantly increased morbidity and mortality with a hazard ratio of 1.9 and a median loss of 5.6 years [4]. In addition to PMNSGCT, various diseases are associated with KS, including male breast cancer, lymphoma, cardiovascular disease, venous thromboembolism, diabetes mellitus, metabolic syndrome, osteoporosis, autoimmune disorders, psychiatric disorders, and neurocognitive disorders [4,11]. Clinicians who are aware of these risks can recommend preventive measures and proper management, which can result in improved quality of life and survival.

Testosterone replacement therapy (TRT) has been the cornerstone of treatment for KS. Hypogonadism is a major contributing factor for most of the physical variations and diseases associated with KS and should be corrected with TRT. Ideally, TRT is recommended to be initiated at the first sign of elevated gonadotropin levels (LH and FSH) even before the onset of low testosterone levels, to allow for proper development of masculine sex characteristics during adolescence and optimize muscle and bone mass while reducing body fat [4,12]. Quality of life, mood, energy, sexual function, and social functioning in KS males are also seen to be improved with TRT. TRT can be given by injection or transdermal gel and is not associated with detrimental side effects in this population. Unfortunately, studies show that TRT is being underutilized. Fertility issues should be addressed before starting TRT, as it can lead to a drop in gonadotropin levels by negative feedback and further reduce spermatogenesis. Despite the majority having azoospermia, KS men can have small foci of spermatozoa in their testicles. MicroTESE is the preferred method and has a success rate of 44-66% [4].

## Conclusions

We presented an uncommon case of concurrent diagnosis and management of PMNSGCT and KS in a young man presenting with a mediastinal mass and physical exam findings consistent with KS. Proper workup and treatment of his PMNSGCT with chemotherapy and surgery resulted in the patient having no evidence of cancer with a high chance of cure. Attention to physical exam findings and knowledge of the association between PMNSGCT and KS allowed for the correct diagnosis of KS, which was missed by physicians who previously cared for him. For his KS, he was referred to multidisciplinary specialists, offered the chance for fertility preservation, and started on testosterone replacement with improvement in various health parameters. We hope the knowledge gained from this report by readers will serve to improve outcomes for patients with PMNSGCT, KS, or both.
